# Correction: Theory for polariton-assisted remote energy transfer

**DOI:** 10.1039/c9sc90224d

**Published:** 2019-11-18

**Authors:** Matthew Du, Luis A. Martínez-Martínez, Raphael F. Ribeiro, Zixuan Hu, Vinod M. Menon, Joel Yuen-Zhou

**Affiliations:** Department of Chemistry and Biochemistry, University of California San Diego La Jolla California 92093 USA joelyuen@ucsd.edu; Department of Chemistry, Department of Physics, Birck Nanotechnology Center, Purdue University West Lafayette IN 47907 USA; Qatar Environment and Energy Research Institute, College of Science and Engineering, HBKU Doha Qatar; Department of Physics, City College, City University of New York New York 10031 USA; Department of Physics, Graduate Center, City University of New York New York 10016 USA

## Abstract

Correction for ‘Theory for polariton-assisted remote energy transfer’ by Matthew Du *et al.*, *Chem. Sci.*, 2018, **9**, 6659–6669.

The authors regret that funding details were incorrect in the Acknowledgements section of the original article. The corrected Acknowledgements section for this article is shown below.

The Royal Society of Chemistry apologises for these errors and any consequent inconvenience to authors and readers.

